# A hidden Markov tree model for testing multiple hypotheses corresponding to Gene Ontology gene sets

**DOI:** 10.1186/s12859-018-2106-5

**Published:** 2018-03-27

**Authors:** Kun Liang, Chuanlong Du, Hankun You, Dan Nettleton

**Affiliations:** 1 Department of Statistics and Actuarial Science, University of Waterloo, Waterloo, N2L 3G1 Canada; 2 Department of Statistics, Iowa State University, Ames, 50011 USA

**Keywords:** Differential expression, Directed acyclic graph, Expectation maximization, Expression quantitative trait loci, False discovery rate, Gene set enrichment analysis

## Abstract

**Background:**

Testing predefined gene categories has become a common practice for scientists analyzing high throughput transcriptome data. A systematic way of testing gene categories leads to testing hundreds of null hypotheses that correspond to nodes in a directed acyclic graph. The relationships among gene categories induce logical restrictions among the corresponding null hypotheses. An existing fully Bayesian method is powerful but computationally demanding.

**Results:**

We develop a computationally efficient method based on a hidden Markov tree model (HMTM). Our method is several orders of magnitude faster than the existing fully Bayesian method. Through simulation and an expression quantitative trait loci study, we show that the HMTM method provides more powerful results than other existing methods that honor the logical restrictions.

**Conclusions:**

The HMTM method provides an individual estimate of posterior probability of being differentially expressed for each gene set, which can be useful for result interpretation. The R package can be found on https://github.com/k22liang/HMTGO.

**Electronic supplementary material:**

The online version of this article (10.1186/s12859-018-2106-5) contains supplementary material, which is available to authorized users.

## Background

An important challenge facing scientists is how to interpret and report the results from high throughput transcriptome experiments, for example, microarray and RNA-seq experiments. Thousands of genes are measured simultaneously from subjects under different treatment conditions. A routine analysis, e.g., a two sample *t*-test for each gene on a microarray, produces a list of genes that are declared to be differential expressed (DE) across conditions. The DE gene list can include hundreds of genes, and this makes the interpretation and reporting of the results a challenging task. However, genes are known to work collaboratively to regulate or participate in biological processes, to perform molecular functions and to produce gene products that form cell components. Thus, it is intuitive and useful to interpret and report results in terms of meaningful gene sets instead of individual genes [1]. It has become a common practice for scientists to test whether some predefined gene categories/sets are differential expressed. Gene Ontology (GO) [2] is one of the most popular sources of gene set definitions. GO provides a controlled vocabulary of terms that form a directed acyclic graph (DAG) with directed edges drawn from general terms to more specific terms. The genes that share a GO term comprise a well-defined gene set. Each GO term and its gene set correspond to a node in the GO DAG. The genes annotated to a specific term are automatically annotated to the more general terms linked by directed edges. Thus, the directed edges also indicate gene set subset relationships. Testing these predefined gene sets on the GO DAG yields meaningful results that are relatively easy to interpret.

Suppose for treatment conditions *c*=1,…,*C* and experimental units *u*=1,…,*n*_*c*_, ***X***_*cu*_ is a vector of *G* gene expression measurements. For *i*=1,…,*N*, suppose ***I***_*i*_ is an indicator matrix such that ***I***_*i*_***X***_*cu*_ is the expression vector for genes in the *i*th GO gene set and the *u*th experimental unit of the *c*th treatment condition. Moreover, suppose that $\boldsymbol {I}_{i} \boldsymbol {X}_{cu}\sim F_{c}^{(i)}$ for all *i*=1,…,*N*; *c*=1,…,*C*; and *u*=1,…,*n*_*c*_. We consider the problem of testing

1 $$  H_{0}^{(i)}: F_{1}^{(i)}=\cdots=F_{C}^{(i)}  $$

for *i*=1,…,*N*. An important goal of biological research is to identify gene sets (or, equivalently, nodes in the GO DAG) for which $H_{0}^{(i)}$ is false (DE nodes) because these are the gene sets whose multivariate expression distribution changes with treatment. Many methods have been proposed to test multivariate gene set differences as in (1), for example, Global Test [3], Global Ancova [4], the Multiple Response Permutation Procedure (MRPP, [5, 6]), Pathway Level Analysis of Gene Expression [7], and Domain-Enhanced Analysis [8], among others.

As a consequence of testing for equality of multivariate distributions within each node of the hierarchical GO DAG, only some configurations of true and false null hypotheses are possible [9–11]. More specifically, if the null hypothesis holds for a gene set *A* then it should hold for all subsets of *A*, which include all the descendants of *A* in a GO DAG. Most of the methods honoring this logical consistency that are applicable to a GO DAG are sequential methods, each of which can be generally classified as a *top-down* or a *bottom-up* procedure [9]. Both procedures are designed to control family-wise error rate (FWER). The top-down procedure based on the closed testing procedure of Marcus et al. [12] is computational prohibitive for large graphs like a GO DAG. Recently, Meijer and Goeman [11] proposed a computationally efficient top-down procedure based on the sequential rejection principle [13]. The bottom-up procedure only tests the leaf nodes of a graph (the nodes without children) and declares significance of some leaf nodes according to a certain FWER control procedure. Then a higher level GO node can be declared significant whenever it has any significant leaf descendant. In the same spirit, the global-up procedure tests all nodes according to a certain FWER control procedure then rejects all ancestors of the rejected nodes. Goeman and Mansmann [9] proposed a focus level method which can be viewed as a combination or compromise between top-down and bottom-up procedures. All sequential methods are subject to power loss due to the fact that a rejection decision has to be made at each step with no regard to the information beyond the current step. For example, if FWER is controlled at the 0.05 level, then a node with a *p*-value of 0.051 will be an impasse for the top-down procedure even if the *p*-value associated with one of its descendant nodes is very small (this could happen when the descendant node has a high concentration of DE genes while the ancestor is “diluted” by many equivalently expressed genes). On the other hand, a DE node’s leaf descendants could all be null nodes, which would render the power for detecting such a DE node to be negligible for a bottom-up procedure.

The structural dependences among null hypotheses can be exploited to make better inferences. Liang and Nettleton [10] proposed a method that circumvents the drawback of the sequential methods by taking the whole graph into account. Their method is fully Bayesian and was shown to have better receiver operating characteristics than other existing methods. However, the implementation of Liang and Nettleton [10] relies on Markov chain Monte Carlo (MCMC) sampling, which can be computationally intensive. There are many circumstances in which a faster approach is needed.

A prime example involves a generalization of expression quantitative trait loci (eQTL) studies. In eQTL studies, a goal is to determine whether variation in DNA at a particular genomic location is associated with variation in the expression of one or more genes. Tens, hundreds, or thousands of genomic locations may be scanned for association with thousands of genes. A natural generalization of eQTL mapping involves testing genomic locations for association with gene sets rather than individual genes. In principle, the approach of Liang and Nettleton [10] could be used for each of many genetic markers to identify associations between markers and traits. However, as the number of markers grows, this strategy quickly becomes computationally intractable. Thus, we develop an alternative and more computationally efficient implementation in this paper.

We present a hidden Markov tree model (HMTM) approach to testing multiple gene sets on a tree-transformed GO DAG. We evaluate its performance through data-driven simulation and an application in the next section.

## Results

### A data-based simulation study

To simulate data that mimics nearly all aspects of real data, we used the simulation procedure proposed by Nettleton et al. [6]. This procedure not only preserves the marginal distribution of genes, but also keeps the correlations among genes largely intact. The dataset of B- and T-cell Acute Lymphocytic Leukemia (ALL) ([14], publicly available through Bioconductor ALL package at www.bioconductor.org) was used in the simulation as a population. The ALL dataset consists of gene expressions of 95 B-cell and 33 T-cell ALL patients measured by Affymetrix HGU95aV2 GeneChips. Ten thousand one hundred seventy seven genes out of the total 12,625 genes measured were mapped to one or more GO terms using hgu95av2.db package version 3.2.3 from Bioconductor, and there were totally 8706 non-empty unique biological process GO terms to be investigated. Note that the electronic annotations (the annotations without the confirmations of human curators) were excluded to increase annotation reliability.

We generate the list of DE genes under two settings. In the first setting, the list of DE genes was derived from the study of Liu et al. [8], who compared their Domain-Enhanced Analysis method using Partial Least Squares with the Fisher’s exact test method on the same ALL dataset and reported a list of the top ten DE gene sets between B- and T-cell patients for each method. We merged the two lists to form a list of 14 unique gene sets. The union of these 14 gene sets consisted of 2435 genes out of the 10177 genes on the GeneChip that were mapped to GO terms. This set of 2435 genes was used to simulate differential expression and will be referred to as the DE gene list. In the second setting, we test each gene set using Global Test [3] and keep the gene sets whose sizes are between 15 and 30 inclusive with *p*-values below 1*e*-6 as our candidate gene sets. The size restriction is to ensure specificity of the candidate gene sets. There are 686 gene sets satisfying the selection criteria, and we randomly choose 40 each time and pool together all genes in theses 40 sets to be the DE genes. The simulation was repeated 200 times under each setting.

**Fig. 1:**
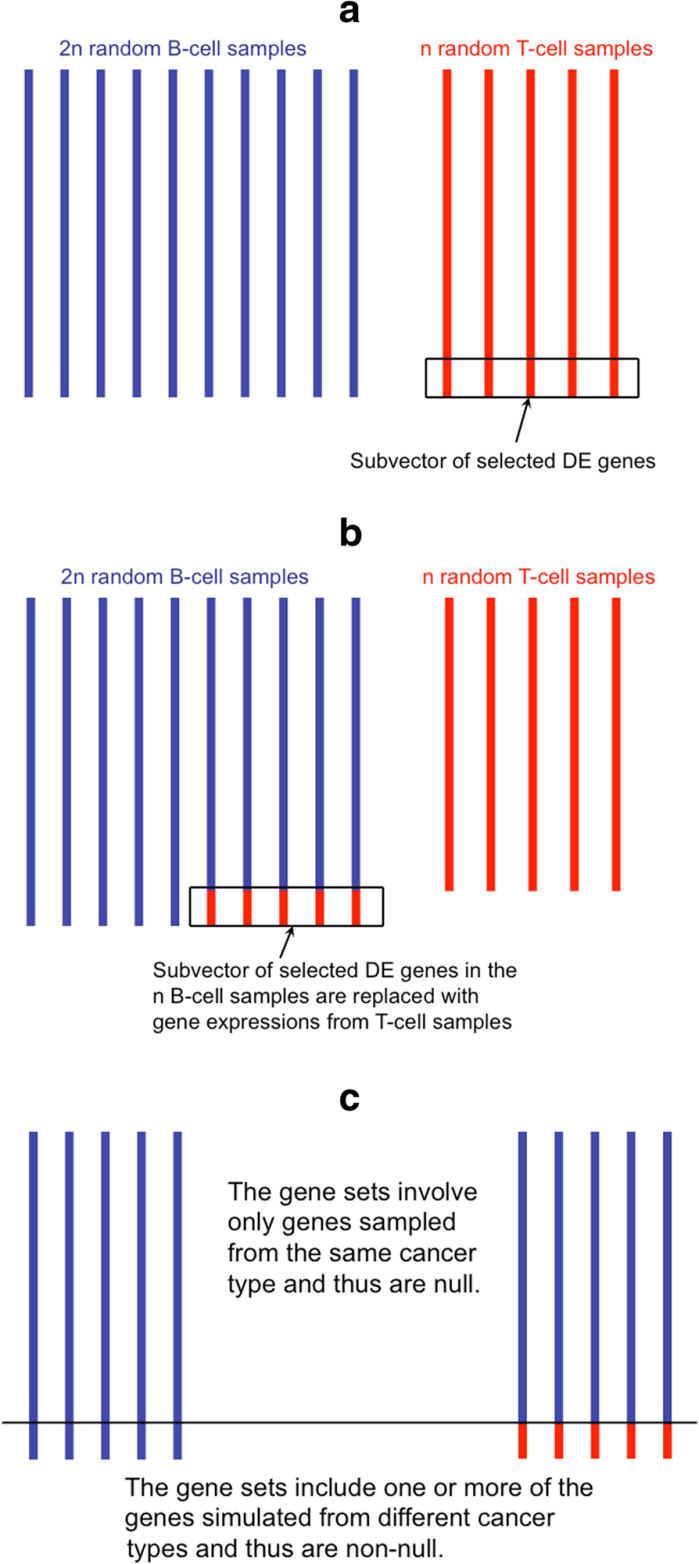
Illustration of the bata-based simulation with ALL dataset and *n*=5

For each simulation run, we generate the dataset as follows: first, 2*n* and *n* patients were drawn randomly without-replacement from B- and T-cell populations, respectively; second, data from the DE genes of the latter half of the 2*n* B-cell patients were replaced with data from the DE genes of the *n* T-cell patients. The first *n* of the B-cell patients were left intact. Then only the 2*n* B-cell patients were kept as our simulated data (*n* intact multivariate observations and *n* modified multivariate observations). The sample of intact observations was then compared to the sample of modified observations. Any gene set containing at least some of the DE genes are DE by construction because the DE genes of the first *n* B-cell patients came from the finite population of 95 B-cell patients, and the DE genes of the latter *n* B-cell patients came from the finite population of 33 T-cell patients. These two finite populations have different mean vectors, different gene-specific variances, different between gene correlations, etc. The null hypotheses corresponding to gene sets containing no DE genes are true nulls by construction because the data vectors corresponding to these gene sets are derived from a random subsample of B-cell patients randomly partitioned into two groups, each of size *n*. An illustration of the data generation steps is shown in Fig. 1. The sample size *n* was chosen to be 9 in our simulation study. The *p*-values of the gene sets could be computed using any of the multivariate gene set testing methods mentioned in the “Background” section. We used the Global Test of Goeman et al. [3], which is based on a score test and is most powerful when many genes have weak effects.

We compared our HMTM method to the top-down procedure of Meijer and Goeman [11] and the global-up procedure, which are described in the “Background” section. The HMTM method was applied to the tree-transformed GO DAG with a probability of differential expression (PDE) significance threshold of 0.99. The latter two methods were applied to the original GO DAG to control FWER at the 0.05 level. The top-down procedure of Meijer and Goeman [11] is implemented in the cherry R package v0.6-11 from the Comprehensive R Archive Network (cran.r-project.org), and we use the any-parent rule, which can be more powerful than the alternative all-parents rule [11].

We also considered other potentially useful methods in our simulation study, but all other methods were ultimately excluded. The min-p procedure proposed by [15] involves permutation of the treatment labels, and it can be computationally demanding. Similarly, the HMM method proposed by [10] was also excluded because of its computational complexity. A small-scale simulation study where the min-p and HMM methods were feasible is included in Additional file 1: Section 2. Another option is the focus level procedure by Goeman and Mansmann [9], but this approach depends much on the choice of a focus level that we have no basis for choosing. Furthermore, the simulation results of Meijer and Goeman [11] show that their top-down procedure has better power performance than the focus level procedure in simulations. Similarly, we excluded the bottom-up procedure because the global-up procedure dominates the bottom-up procedure in terms of power and the receiver operating characteristic in our simulation settings.

As shown in Table 1, both FWER-controlling methods exhibited excellent performance with regard to type I error control. Few type I errors were made by either of the FWER-controlling methods across all 200 simulated datasets. The top-down procedure had poor power in setting 2 because the DE gene sets are relatively small and far from the root node. The HMTM method exhibited far more power than either of the FWER-controlling methods, identifying more than twice as many true positive results at the cost of a modest number of false positives on average, relative to the number of discoveries.

**Table 1:** Average number of rejections and false positives across 200 simulated datasets for the proposed HMTM method, top-down procedure, and global-up procedure. *R* denotes # of rejections; *V* denotes # of false positives

	HMTM			Top-down			Global-up	
Setting	*R*	*V*		*R*	*V*		*R*	*V*
1	2515	1.2		1077	0.005		1061	0.01
2	2538	28.4		75	0.01		595	0.005

**Fig. 2:**
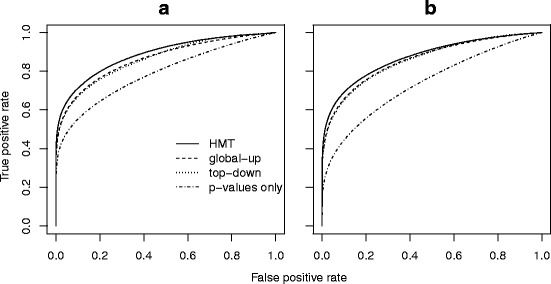
ROC curves for HMTM, global-up, top-down and *p*-values only methods in simulation results. Panel **a**: setting 1; **b**: setting 2

Because different methods use different error rates, it is important to examine the trade-off between sensitivity and specificity in each case. To allow a fair comparison and further illustrate the advantage of the newly developed HMTM method, we used receiver operating characteristic (ROC) curves in Fig. 2 to compare the HMTM method with the other two methods and a method based only on *p*-values. The latter method rejects the GO DAG nodes by their *p*-value in an ascending order without regard to the GO DAG structure.

It is clear from Fig. 2 that the *p*-values only method performs the worst because it completely ignores all GO DAG structural information. The performance of top-down and global-up procedures are similar. The HMTM method achieves the best performance because it fully utilizes the GO DAG structural information by modeling the whole GO DAG. Thus, the power advantage exhibited in our Table 1 simulation result was not simply a consequence of differing error control criteria. By modeling the structural dependence among the null hypotheses, the HMTM method turns the restrictions on the GO DAG into information and is superior to the methods simply ignoring the information or the methods passively obeying the restrictions.

### Application to eQTL data

Our HMTM method was applied to a large-scale expression quantitative trait loci (eQTL) dataset collected by West et al. [16]. Quantitative trait loci (QTL) studies are conducted to discover the locations of genotype variants that explain the expression variations for a particular gene. In eQTL studies, the expression levels of thousands of genes are measured simultaneously by microarray or RNA sequencing, and the locations of genotype variants affecting each gene are searched. The dataset contains 211 recombinant inbred lines (RIL) of Arabidopsis thaliana, a model organism in plant genetics. Each RIL was measured on two biological replicates, and a total of 422 Affymetrix ATH1 GeneChips were used. Each GeneChip measures 22,810 genes of Arabidopsis thaliana. The microarray dataset can be obtained at http://elp.ucdavis.eduMicroarray measurements were normalized using the robust multichip average (RMA) method [17]. The measurements of the two biological replicates were averaged to give a single transcript measurement per gene and RIL.

These 211 RILs are part of a population of 420 RILs that were genotyped by Loudet et al. [18]. The 420 RILs are the result of crossing between two genetically distant ecotypes, Bay-0 and Shahdara. A set of 38 physically anchored microsatellite markers were measured for each RIL, and the genotype at each marker either comes from Bay-0 or Shahdara.

Traditional eQTL studies scan the expression data of each gene against a large number of genotyped locations and can easily have millions of hypotheses being tested. We hypothesize that by testing the genotype effect on gene sets instead of genes, we could potentially reduce the burden of multiplicity adjustment and increase the power of signal detection. Using version 3.2.3 of the ath1121501.db Bioconductor package, 3108 unique non-empty GO terms from the biological process ontology were identified. The goal of our analysis is to test for association between marker genotypes and gene set expression vectors corresponding to these GO terms. The *p*-values for the gene sets corresponding to the GO tree nodes were computed using the Global Test method [3]. For each of the 38 markers, the HMTM method was carried out to calculate the PDEs for the GO terms.

**Fig. 3:**
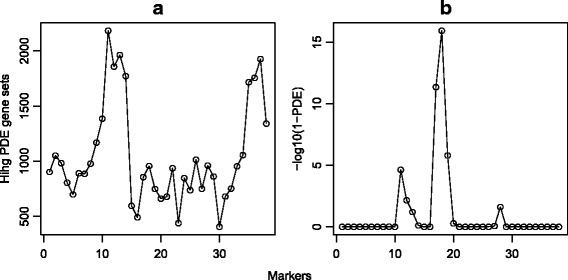
Analysis of the eQTL dataset from West et al. [16]. Panel **a**: Number of high PDE gene sets; **b**: PDEs of “GO:0031117”

To our best knowledge, this is the first systematic testing of GO terms as a structured multiple testing problem in the eQTL setting. Figure 3a shows the number of high PDE gene sets (PDE >0.999) across markers and suggests markers 11–14 and 35–37 are the most active markers in regulating biological processes. The results associated with Fig. 3b illustrate why our HMTM method is more powerful than the sequential FWER-controlling top-down procedure. PDEs of GO term “GO:0031117”, positive regulation of microtubule depolymerization, were plotted against markers. It is evident that there is an eQTL for the gene set near marker 17 and 18. The Global Test *p*-values for the GO term at the two markers are 1.7e-7 and 4.5e-13, respectively. On the other hand, one of its ancestor GO terms, “GO:0051130”, has *p*-values of 0.30 and 0.28 at the two markers. If the top-down procedure were used, the highly significant GO term “GO:0031117” would never be tested even at an FWER level of 0.2.

## Discussion

Although we use an empirical null to accommodate the dependencies among null *p*-values in our HMTM method, the dependence structure among overlapping gene sets is complex, and the control of FDR cannot be guaranteed. On the other hand, FWER-controlling methods provide the control of FWER despite dependence. We would recommend that practitioners use any FWER control method as a first step. If the FWER method declares that no gene set is DE, then stop and reject nothing. Otherwise, our HMTM method can be applied. This added step will provide weak control of FWER, i.e., control of FWER when all the null hypotheses are true. Note that none of the results in our paper would change with this modification.

By testing multivariate distributional difference of gene sets as in (1), all gene sets that contain DE genes are considered DE. For a particular genetic experiment, there could be a large number of DE gene sets declared, among which many share the same DE genes due to gene set overlap. To address the difficulty to interpret many overlapping DE gene sets, Bauer et al. [19] developed the model-based gene set analysis (MGSA) methodology to identify a short list of gene sets that provide parsimonious explanation for the observed DE gene status. Assuming a list of DE genes is available, they model the probability of a gene belongs to the DE gene list as a simple function of whether the gene belongs to any DE gene sets. For identifiability reasons, Newton et al. [20] further assumes that all genes in the DE gene sets are DE, and Wang et al. [21] developed the corresponding computationally efficient methods applicable to large-scale gene set testing.

Although it is appealing to have fewer and more representative DE gene sets, the MGSA methods also have drawbacks. By modeling only a list of DE genes, the MGSA methods are oblivious to other information, such as the test statistics of all genes. Furthermore, the list of DE genes is typically compiled by marginally testing each gene for differential expression and reporting the top genes with the smallest *p*-values. If the list of DE genes is obtained through marginal testing, the MGSA methods may have little power to detect the multivariate distributional difference of a set of genes or gene sets with weak but consistent individual gene effects [6, 9]. Combining the power of the multivariate distribution testing and the interpretation advantage of the model-based methods could be an interesting future research direction.

## Conclusion

When testing multivariate distributional difference in gene sets on the GO DAG, our HMTM method provides a more powerful and sensible solution than the existing sequential methods. The improved power comes from our method’s ability to borrow information throughout the GO DAG structure. The ROC curves in Fig. 2 show that our method was better able to distinguish DE gene sets from equivalently expressed gene sets than existing methods. Furthermore, our HMTM method provides an individual estimate of posterior probability of being DE for each gene set/hypothesis, while the FWER-controlling methods only return a set of rejected hypotheses given a specific FWER threshold.

The HMTM method is also more computationally efficient than the HMM method proposed by Liang and Nettleton [10], and the reduction of computation time can be substantial. For example, to analyze the simulated datasets in the “” section, the HMM method of Liang and Nettleton [10] would consume about 50 h for each dataset while the HMTM method requires less than 2 min. This is a reduction of computation time for more than three orders of magnitude. Thus, the proposed HMTM method is both powerful in inference and efficient in computation.

## Methods

The logical constraints among the null hypotheses on a GO DAG induce a natural Markov model on the states of the null hypotheses, but exact computation on a complex graph like the GO DAG is computationally prohibitive [10]. Thus, following Liang and Nettleton [10], we transform a GO DAG into a GO tree to facilitate the computation. Then, a single *p*-value for testing the null hypothesis in (1) is computed separately for each node in the GO tree. We then model the joint distribution of these tree node *p*-values using a hidden Markov tree model. We treat the state of each null hypothesis as a random variable and propose a Markov model for the joint distribution of states. This Markov model places zero probability on any configuration of states that is not consistent with the logical constraints imposed by the structure of the GO tree.

We summarize the tree transformation and hidden Markov model in Liang and Nettleton [10] in the following two subsections. Then we use a hidden Markov tree model to obtain the maximum likelihood estimates of the parameters. Furthermore, instead of sampling state configurations given the parameters, we deterministically compute the probabilities of the original DAG nodes being DE. Thus, the new implementation dramatically reduces the computational expense of the estimation process.

### Tree transformation of a GO DAG

**Fig. 4:**
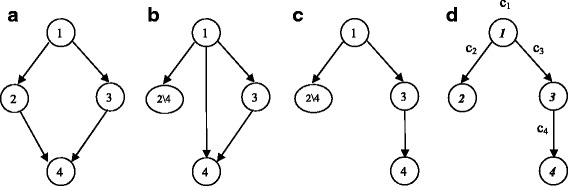
DAG to tree transformation: **a** Original DAG; **b** After remove genes in node 4 from node 2; **c** Tree after remove redundant edge from node 1 to node 4; **d** Tree nodes renumbered with bold and italic numbers

Transforming a GO DAG into a tree structure can make computation feasible on one hand and greatly reduce the sharing of genes and dependences among gene sets on the other hand. The tree transformation process is illustrated using a tiny example in Fig. 4. Interested readers can refer to Section 3.1 of Liang and Nettleton [10] for a more detailed description of the process. The basic idea of the tree transformation is as follows. If we remove all but one incoming edges for each node that has multiple parents, the graph becomes a tree. This is equivalent to removing the genes in the child node from all but one of its parent nodes. For example, see the removal of the edge from node 2 to 4 in Fig. 4a.

After the procedure, every node except the root node will have one and only one parent, and thus, the DAG will be transformed into a tree. Each of the original DAG nodes will be a union of one or more tree nodes. For example, DAG node 2 in Fig. 4a is a union of tree nodes ***2*** and ***4*** in Fig. 4d. More formally, for *j*=1,…,*N*_*G*_; let $\mathcal {G}_{j}$ be the gene set corresponding to GO DAG node *j*. For *i*=1,…,*N*_*T*_; let $\mathcal {T}_{i}$ be the set of genes that are in GO tree node *i*. Let $\mathcal {G T}_{j}$ denote the set of tree nodes/indices whose corresponding gene sets are subsets of $\mathcal {G}_{j}$, i.e., $\mathcal {G T}_{j} = \{k=1,\ldots,N_{T}: \mathcal {T}_{k} \subseteq \mathcal {G}_{j}\}$. The tree transformation process guarantees that the original DAG node can be reconstructed from its comprising tree nodes, i.e., $\mathcal {G}_{j} = \bigcup _{k \in \mathcal {G T}_{j}} \mathcal {T}_{k}$. Let the state of *i*th GO tree node be *S*_*i*_. Let *S*_*i*_=0 if $H_{0}^{(i)}$ is true and let *S*_*i*_=1 if $H_{0}^{(i)}$ is false. For the *j*th GO DAG node, define

2 $$\begin{array}{@{}rcl@{}} S^{*}_{j} = \max\left\{S_{k}: k \in \mathcal{G T}_{j}\right\}. \end{array} $$

Note that $S^{*}_{j} = 1$ implies that the state of GO DAG node *j* is 1 because a vector of genes corresponding to a gene set must have different multivariate distributions across treatment conditions if any subvector does. It is straightforward to show this conversion guarantees the logical consistency of states $\left \{S^{*}_{j}: j=1,\ldots,N_{G}\right \}$ for the original GO DAG. In the end of this section, we will show how to estimate, for *j*=1,…,*N*_*G*_, the probability that $S^{*}_{j}=1$ using the results derived from a HMTM on the corresponding GO tree.

### A hidden Markov tree model for *p*-values on the GO Tree

By the nature of the null hypothesis of multivariate distribution equivalence in (1) and the subset relationship among GO tree gene sets, a node must be in state 0 if its parent node is in state 0. On the other hand, a node whose parent is in state 1 can be in state 1 with some unknown probability. This conditional dependence scenario clearly demonstrates the Markov property.

Thus, the hidden Markov tree model (HMTM) is proposed as follows. Let *S*_*i*_ be the state of *i*th GO tree node as defined before, and let *p*_*i*_ be the *p*-value associated with GO tree node *i* (gene set *i*) that is computed by testing (1) using any method that produces a valid *p*-value. Then the HMTM involves an observed random tree $\boldsymbol {p} = \left \{p_{1}, \ldots, p_{N_{T}}\right \}$ and an unobserved random tree $\boldsymbol {S} = \left \{S_{1}, \ldots, S_{N_{T}}\right \}$. Both trees have the same index structure. Let *ρ*(*i*) denote the index of the parent node of node *i*. The transition portion of our HMTM is

3 $$  \text{P}\left(S_{i}=0|S_{\rho(i)}=0\right) = 1 ~ \text{and} ~ \text{P}(S_{i}=1|S_{\rho(i)}=1) = \omega,  $$

for some *ω*∈(0,1). To streamline the expressions of recursion in the future, we express (3) in an equivalent way through the generic definition of transition probabilities. Let *q*_*jk*_=P(*S*_*i*_=*k*|*S*_*ρ*(*i*)_=*j*) be the transition probability from a parent node in state *j* to a child node in state *k*, and thus, *q*_00_=1,*q*_01_=0,*q*_10_=1−*ω* and *q*_11_=*ω*. Furthermore, we assume the root node of the tree (the node with no parent) is in state 1 with some probability *π*∈(0,1). To model the observed *p*-values given the hidden states, we consider the model

4 $$  {{} \begin{aligned} \left\{ \begin{array}{ll} p_{i} \sim f_{0}(\lambda, \alpha_{0}, \beta_{0}) = \lambda+(1-\lambda)\text{beta}(\alpha_{0}, \beta_{0}) &~\text{if}~ S_{i}=0\\ p_{i} \sim f_{1}(\alpha, \beta) = \text{beta}(\alpha, \beta) &~\text{if}~ S_{i}=1 \end{array} \right. \end{aligned}}  $$

with *p*-values assumed to be conditionally independent of one another given the states. The conditional independence assumption is clear false because gene sets share genes, and we use a mixture model under the null to accomodate the potential dependence. More specifically, the *p*-value density of true nulls is assumed to be a mixture of uniform and unimodal beta, where *λ* denotes the mixing proportion. The parameters *α*_0_ and *β*_0_ are restricted to be bigger than 1 so that a unimodal *p*-value density is guaranteed. Notice that a uniform model or a unimodal beta model is a degenerated case of this mixture model. In most cases, a simple uniform model will work well. However, the null mixture model is designed to adapt to the possible deviation from the uniform distribution caused by positive correlations among the null gene sets due to the sharing of genes and correlations among genes. This alteration of the commonly used uniform null *p*-value distribution is similar in spirit to the approach of Efron [22] who recommends using data to estimate an “empirical” null distribution. The parameters *α* and *β* for the *p*-value density of false nulls are restricted to be in (0,1] and (1,*∞*), respectively, so that a strictly decreasing *p*-value density is guaranteed for DE gene sets.

Let ***θ***={*π*,*ω*,*α*,*β*,*λ*,*α*_0_,*β*_0_}, the collection of all HMTM parameters. Liang and Nettleton [10] used a Bayesian approach that assumes ***θ*** to be random with diffuse priors. To speed up the estimation, we assume in this paper that ***θ*** is a vector of fixed unknown parameters to be estimated. In essence, we are using an empirical Bayes approach instead of the fully Bayesian approach, and the two approaches are expected to give similar results when the GO tree contains many nodes.

### Upward-downward Algorithm for HMTM

The forward-backward algorithm is widely used in hidden Markov chain applications; its parallel in hidden Markov tree models is the upward-downward algorithm developed by Ronen et al. [23] and Crouse et al. [24]. Durand et al. [25] reformulated the algorithm to make the algorithm numerically stable. Given the parameter vector ***θ***, the upward-downward algorithm leads to efficient computation of the likelihood, $\mathcal {L}(\boldsymbol {\theta }|\boldsymbol {p})$. Furthermore, the results from the upward-downward algorithm are useful in obtaining the maximum likelihood estimates of parameters in the next subsection and computing probabilities of differential expression of the nodes on the original GO DAG in the last subsection. We formulate our HMTM on the GO tree in the framework of Durand et al. [25] as follows.

Without loss of generality, let the root node of the GO tree be indexed by 1. Let *i*=1,…,*N*_*T*_ be any GO tree node index and *k*=0 or 1 be a possible state of a node. Let ***C***(*i*) denote the set of indices of node *i*’s children nodes. Let $\mathfrak {T}(i)$ denote the subtree whose root is node *i*. Let ***p***_*i*_ be a vector of *p*-values corresponding to the subtree rooted at node *i*, i.e., ***p***_*i*_ is a vector whose elements are $\{p_{l}: l \in \mathfrak {T}(i)\}$. Denote ***p***_*i*∖*j*_ as a vector of *p*-values corresponding to the nodes in subtree $\mathfrak {T}(i)$ but not in $\mathfrak {T}(j)$, i.e., ***p***_*i*∖*j*_ is a vector whose elements are $\{p_{l}: l \in \mathfrak {T}(i); l \notin \mathfrak {T}(j)\}$. Let *f*(·) and *f*(·|·) denote a generic density and conditional density, respectively, whose precise definitions are easily inferred from function arguments. Assuming ***θ*** is known, we define three quantities that can be computed efficiently by recursion:

$$\begin{array}{@{}rcl@{}} \tau_{i}(k) &=& \text{P}\left(S_{i}=k|\boldsymbol{p}_{i}\right);\\ \tau_{\rho(i), i}(k) &=& \frac{f\left(\boldsymbol{p}_{i}|S_{\rho(i)}=k\right)}{f(\boldsymbol{p}_{i})};\\ \kappa_{i}(k) &=& \frac{f\left(\boldsymbol{p}_{1 \backslash i}|S_{i}=k\right)}{f(\boldsymbol{p}_{1 \backslash i}|\boldsymbol{p}_{i})}. \end{array} $$

First we compute the marginal state probabilities P(*S*_*i*_=*k*) for *i*=1,…,*N*_*T*_ and *k*=0 or 1 in a downward recursion, i.e., P(*S*_1_=*k*)=*π*^*k*^(1−*π*)^1−*k*^ and $\text {P}(S_{i}=k) = \sum _{j} q_{jk}\text {P}\left (S_{\rho (i)}=j\right)$ for *i*>1. Then the *τ*_*i*_(*k*) quantities can be computed recursively in an upward fashion. For any leaf node *i*, *τ*_*i*_(*k*) is initialized as

$$\begin{array}{@{}rcl@{}} \tau_{i}(k) = \frac{f(p_{i}|S_{i}=k)\text{P}(S_{i}=k)}{N_{i}}, \end{array} $$

where $N_{i} = \sum _{k} f(p_{i}|S_{i}=k)\text {P}(S_{i}=k)$ is a normalizing factor for the leaf node *i* such that $\sum _{k} \tau _{i}(k)=1$. An upward computation for a non-leaf node is

$$\begin{array}{@{}rcl@{}} \tau_{i}(k) = \frac{f(p_{i}|S_{i}=k)\text{P}(S_{i}=k)\prod_{\nu \in {\mathcal{C}}(i)} \tau_{i, \nu}(k)}{N_{i}}, \end{array} $$

where $\phantom {\dot {i}\!}N_{i}=\sum _{k=0}^{1} \left [f(p_{i}|S_{i}=k)\text {P}(S_{i}=k)\prod _{\nu \in \mathcal {C}(i)} \tau _{i, \nu }(k) \right ]$ is the normalizing factor for the non-leaf node. The *τ*_*ρ*(*i*),*i*_(*k*) quantities can be derived from the *τ*_*i*_(*k*)s as follows:

$$\begin{array}{@{}rcl@{}} \tau_{\rho(i), i}(k) = \sum\limits_{j} \frac{\tau_{i}(j)q_{kj}}{\text{P}(S_{i}=j)}. \end{array} $$

Note that the upward recursion process requires us to compute *τ*_*i*_(*k*)s for the leaf nodes first, then *τ*_*ρ*(*i*),*i*_(*k*)s for the leaf nodes, then *τ*_*i*_(*k*)s for the parents of the leaf nodes, and so forth.

The *κ*_*i*_(*k*) quantities are computed in a downward fashion. After we initialize *κ*_1_(0)=*κ*_1_(1)=1, the downward recursion is

$$\begin{array}{@{}rcl@{}} \kappa_{i}(k) = \frac{1}{P(S_{i}=k)} \sum\limits_{j} \frac{q_{jk} \tau_{\rho(i)}(j)\kappa_{\rho(i)}(j)}{\tau_{\rho(i), i}(j)}. \end{array} $$

It can be shown that the log-likelihood $l(\boldsymbol {\theta }|\boldsymbol {p}) = \sum _{i} \log N_{i}$, which is useful for monitoring the convergence of the expectation maximization (EM) algorithm in the next subsection.

### EM Algorithm

The EM algorithm [26] is commonly used for estimating the parameters of a hidden Markov model. For example, the widely used Baum-Welch algorithm [27] is a special case of the EM algorithm. We will show how to find $\hat {\boldsymbol {\theta }} = \underset {\boldsymbol {\theta }}{\arg \!\max } ~ l(\boldsymbol {\theta }|\boldsymbol {p})$, the maximum likelihood estimate of ***θ***, through EM.

For the E step of the EM algorithm,

$$\begin{array}{@{}rcl@{}} Q(\boldsymbol{\theta}|\boldsymbol{\theta}^{(t)}) &=& \mathrm{E}_{\S|\boldsymbol{p}, \boldsymbol{\theta}^{(t)}} \left[\log {\mathcal L}(\boldsymbol{\theta}|\boldsymbol{p}, \boldsymbol{S})\right]\\ &=& \mathrm{E}_{\boldsymbol{S}|\boldsymbol{p}, \boldsymbol{\theta}^{(t)}} \left[{\vphantom{\sum\limits_{i=2}^{N_{T}}}}S_{1} \log \pi + (1-S_{1})\log (1-\pi)\right. \\ & & +\sum\limits_{i=2}^{N_{T}} \textup{I}(S_{\rho(i)}=1, S_{i}=1) \log \omega \\ & & +\sum\limits_{i=2}^{N_{T}} \textup{I}(S_{\rho(i)}=1, S_{i}=0) \log (1-\omega) \\ & &+ \sum\limits_{i=1}^{N_{T}} S_{i} \log f_{1}(p_{i}|\alpha, \beta)\\ && +\left. \sum\limits_{i=1}^{N_{T}} (1-S_{i}) \log f_{0}(p_{i}|\lambda, \alpha_{0}, \beta_{0})\right]. \end{array} $$

In the *Q*(***θ***|***θ***^(*t*)^) expression, the conditional expectations for the terms associated with *S*_*i*_s can be derived separately as follows:

$${{} \begin{aligned} \mathrm{E}\left(S_{i}|\boldsymbol{p}, \boldsymbol{\theta}^{(t)}\right) = \text{P}\left(S_{i}=1|\boldsymbol{p}, \boldsymbol{\theta}^{(t)}\right) &= \tau_{i}^{(t)}(1) \kappa_{i}^{(t)}(1);\\ \mathrm{E}\left[\text{I}\left(S_{\rho(i)}=1, S_{i}=1\right)|\boldsymbol{p}, \boldsymbol{\theta}^{(t)}\right] &= \frac{\tau_{i}^{(t)}(1) \omega^{(t)} \mathrm{E}\left(S_{\rho(i)}|\boldsymbol{p}, \boldsymbol{\theta}^{(t)}\right)} {\text{P}(S_{i}=1)\tau_{\rho(i), i}^{(t)}(1)};\\ \mathrm{E}\left[\text{I}\left(S_{\rho(i)}\,=\,1, S_{i}\,=\,0\right)|\boldsymbol{p}, \boldsymbol{\theta}^{(t)}\right] &= \frac{\tau_{i}^{(t)}(0)\left(1-\omega^{(t)}\right) \mathrm{E}\left(S_{\rho(i)}|\boldsymbol{p}, \boldsymbol{\theta}^{(t)}\right)} {\text{P}(S_{i}=0)\tau_{\rho(i), i}^{(t)}(1)}.\\ \end{aligned}} $$

In the M step, we obtain $\boldsymbol {\theta }^{(t+1)} = \underset {\boldsymbol {\theta }}{\arg \!\max } ~ Q\left (\boldsymbol {\theta }|\boldsymbol {\theta }^{(t)}\right)$. Let $\text {P}_{1k} = \sum _{i=2}^{N_{T}} \mathrm {E}\left [\text {I}\left (S_{\rho (i)}=1, S_{i}=k\right)|\boldsymbol {p}, \boldsymbol {\theta }^{(t)}\right ]$, *k*=0 or 1. By solving score functions, we have

$$\begin{array}{@{}rcl@{}} \pi^{(t+1)} &=& \mathrm{E}\left(S_{1}|\boldsymbol{p}, \boldsymbol{\theta}^{(t)}\right),\\ \text{and}~ \omega^{(t+1)} &=& \frac{\text{P}_{11}}{\text{P}_{11}+\text{P}_{10}}. \end{array} $$

The parameters *α* and *β* can be estimated by numerically maximizing a sum of weighted log-likelihoods given by $\phantom {\dot {i}\!}\sum _{i=1}^{N_{T}} w_{i} \log f_{1}(p_{i}|\alpha, \beta)$, where *w*_*i*_=E(*S*_*i*_|***p***,***θ***^(*t*)^) for *i*=1,…,*N*_*T*_. The parameters *λ*,*α*_0_ and *β*_0_ can be estimated similarly.

However, the EM result can highly depend on its initial parameter values especially in a multivariate context like ours. We use two methods to alleviate the dependence on the initial value. The first method is to perform EM from many (different) random starting values. The second method is the deterministic annealing (DA) method through the principle of the maximum entropy [28]. The detail of adapting the DA method to our problem can be found in the Additional file 1: Section 1. In practice, we use both methods and keep the result from the one with larger likelihood.

### Compute state probabilities for the original GO DAG nodes

At the end, the results on the GO tree need to be converted back to the state probabilities on the original GO DAG. We design an efficient algorithm to do so through the use of conditional transition probabilities on the GO tree. Define *c*_*jk*_(*i*) as the probability of GO tree node *i* being state *k* conditional on all the observed data (***p***) and its parent being in state *j*. Given ***θ*** and for *i*=2,…,*N*_*T*_, *c*_*jk*_(*i*)s can be computed from the upward probabilities as follows:

5 $$\begin{array}{@{}rcl@{}} c_{jk}(i) & \equiv & \text{P}\left(S_{i}=k|\boldsymbol{p}, S_{\rho(i)}=j\right)  \\ & = & \text{P}\left(S_{i}=k|\boldsymbol{p}_{i}, S_{\rho(i)}=j\right)  \\ & = & \frac{f\left(S_{i}=k, \boldsymbol{p}_{i}|S_{\rho(i)}=j\right)}{f\left(\boldsymbol{p}_{i}|S_{\rho(i)}=j\right)}  \\ & = & \frac{f(\boldsymbol{p}_{i}|S_{i}=k)\text{P}(S_{i}=k|S_{\rho(i)}=j)}{f(\boldsymbol{p}_{i}|S_{\rho(i)}=j)}  \\ & = & \frac{q_{jk} \text{P}(S_{i}=k|\boldsymbol{p}_{i}) f(\boldsymbol{p}_{i}) / \text{P} (S_{i}=k) }{f(\boldsymbol{p}_{i}|S_{\rho(i)}=j)}  \\ & = & \frac{q_{jk} \tau_{i} (k)}{\tau_{\rho(i), i}(j) \text{P}(S_{i}=k)}. \end{array} $$

To simplify the notation for our two-state GO tree, define *c*_*i*_≡*c*_11_(*i*). By logical restriction, *c*_00_(*i*)=1, and *c*_01_(*i*)=0. Furthermore, *c*_10_(*i*)=1−*c*_11_(*i*), so *c*_*i*_ is sufficient for computation of all four conditional transition probabilities. Thus, from (5) and for *i*=2,…,*N*_*T*_,

6 $$\begin{array}{@{}rcl@{}} c_{i} = \frac{\omega \tau_{i} (1)}{\tau_{\rho(i), i}(1) \text{P}(S_{i}=1)}.  \end{array} $$

Finally, it is straightforward to show that *c*_1_=*τ*_1_(1). Our derivation of *c*_*i*_’s has not been shown in literature before, but the result is very useful in applications.

Recall that the state of *j*th GO DAG node $S^{*}_{j} = \max \left \{S_{k}: S_{k} \in \mathcal {G T}_{j}\right \}$, i.e., the maximum of its comprising tree node states. Given ***θ***, define $\text {PDE}_{j} = \text {P}_{\boldsymbol {\theta }}\left (S^{*}_{j}=1|\boldsymbol {p}\right)$, the conditional probability that the *j*th GO DAG node is in state 1 (or, equivalently, that gene set $\mathcal {G}_{j}$ is DE) given all *p*-values corresponding to nodes of the HMTM on the GO tree as defined before. It is straightforward to use *c*_*i*_s to compute the PDE_*j*_s by using the GO tree structure and conditional independence of the states in the HMTM. For example, in the toy example in Fig. 4, original GO DAG node 2 is the union of tree nodes 2 and 4. Then the probability that DAG node 2 is in state 1 is the probability that either tree node 2 or 4 is in state 1. Note that *S*_2_ and *S*_4_ are independent given *S*_1_ and ***p***. Furthermore, *c*_*i*_s are computed as in (6) and annotated in Fig. 4d. Then the computation can be carried out as follows:

$${{}\begin{aligned} \text{PDE}_{2} &= \text{P}(S^{*}_{2}=1|\textup{HMTM})\\ &= \text{P}(S_{2}=1 ~\text{or}~ S_{4}=1|\boldsymbol{p})\\ &= \text{P}(S_{1}=1|\boldsymbol{p})\text{P}(S_{2}=1 ~\text{or}~ S_{4}=1|S_{1}=1,\boldsymbol{p})\\ &= \text{P}(S_{1}=1|\boldsymbol{p})\left[1-\text{P}(S_{2}=0, S_{4}=0|S_{1}=1,\boldsymbol{p})\right]\\ &= \text{P}(S_{1}=1|\boldsymbol{p})\left[1-\text{P}(S_{2}=0|S_{1}=1,\boldsymbol{p})\text{P}(S_{4}=0|S_{1}=1,\boldsymbol{p})\right]\\ &= c_{1} [1-(1-c_{2})(1-c_{3} c_{4})]. \end{aligned}} $$

The second from the last step is due to the fact that *S*_2_ and *S*_4_ are independent given *S*_1_ and ***p***. The PDEs of each GO DAG node can be carried out in similar way with tedious technical computations. We estimate ***θ*** as $\hat {\boldsymbol {\theta }}$ as in the previous subsection, then compute the plug-in estimates of $\hat {c}_{i}$s and $\widehat {\text {PDE}}_{j}$s using $\hat {\boldsymbol {\theta }}$.

### Rejection region

By definition, $\phantom {\dot {i}\!}1-\text {PDE}_{i} = \text {P}_{\boldsymbol {\theta }}\left (S^{*}_{j}=0|\boldsymbol {p}\right)$, which is closely related to the local index of significance defined by Sun and Cai [29] in their work on testing HMM-dependent hypotheses. For any rejection index set *R*, a natural estimate for the FDR is

7 $$  1-\frac{1}{|R|} \sum\limits_{i \in R} \widehat{\text{PDE}}_{i},  $$

i.e., 1 − the average of the PDE estimates for nodes in the rejection set. However, as noted by Goeman and Mansmann [9] and Liang and Nettleton [10], FDR may not be an appropriate quantity to control in a structured hypothesis testing problem like the GO DAG. Thus, we recommend selecting a subset of nodes with the highest estimated PDE values with suggested threshold for significance of 0.95 or 0.99, for example.

## Additional file

Additional file 1 Supplementary Material. Details of deterministic annealing and additional simulation result. (PDF 152 kb)

## Abbreviations

ALL: Acute lymphocytic leukemia
DAG: Directed acyclic graph
DE: Differential expressed
eQTL: Expression quantitative trait loci
FWER: Family-wise error rate
GO: Gene Ontology
HMTM: Hidden Markov tree model
MCMC: Markov chain Monte Carlo
PDE: Probability of differential expression
ROC: Receiver operating characteristic
