# Supplementary material for: A hidden Markov tree model for testing multiple hypotheses corresponding to Gene Ontology gene sets
Source: BMC Bioinformatics. 2018 Mar 27;19:107. doi: 10.1186/s12859-018-2106-5 (PMC5869792; doi:10.1186/s12859-018-2106-5)
Supplement: Supplementary file 1 — Supplementary Material. Details of deterministic annealing and additional simulation result. (PDF 152 kb) [file 12859_2018_2106_MOESM1_ESM.pdf]

Supplementary Material for "A Hidden Markov Tree Model for Testing Multiple Hypotheses Corresponding to Gene Ontology Gene Sets"

May 25, 2017

In Section 1, we describe the adaptation details of the deterministic annealing method in the EM algorithm to complement Section 3.4 of the main text. In Section 2, we performed a small-scale simulation study to compare the proposed HMTM method with the HMM method of Liang and Nettleton (2010), the min-p procedure of Westfall and Young (1993) and other procedures.

1. DETERMINISTIC ANNEALING

Ueda and Nakano (1998) proposed a deterministic annealing EM (DAEM) algorithm to alleviate EM's dependency on starting values. Through the principle of the maximum entropy, they derived a posterior that is parameterized by a "temperature" parameter, which is used to control an "annealing process".

Adapting the DAEM framework to our HMTM problem, the posterior of the unobserved/missing data $\boldsymbol{S}$ is

$$f_\gamma(\boldsymbol{S}|\boldsymbol{p},\boldsymbol{\theta}) = \frac{f^\gamma(\boldsymbol{p},\boldsymbol{S}|\boldsymbol{\theta})}{\int f^\gamma(\boldsymbol{p},\boldsymbol{S}|\boldsymbol{\theta})\ \mathrm{d}\boldsymbol{S}},$$

where $1/\gamma$ corresponds to the "temperature." Note that if $\gamma = 1$ then the posterior is exactly the same as a regular posterior. On the other hand, when γ is close to 0 (temperature is high), $f^\gamma(\boldsymbol{p},\boldsymbol{S}|\boldsymbol{\theta})$ is close to 1 and is insensitive to the values of $\boldsymbol{p}$ and $\boldsymbol{\theta}$. So if we start with a high temperature, the impact of initial parameter values will be minimized. The DAEM operates according to a deterministic schedule of temperatures, in which the temperature drops from high to low (γ changes from near zero to 1). At each temperature/γ, an EM algorithm is used to estimate $\boldsymbol{\theta}$ assuming the conditional distribution of $\boldsymbol{S}$ given $\boldsymbol{p}$ and $\boldsymbol{\theta}$

follows $f_\gamma(\boldsymbol{S}|\boldsymbol{p},\boldsymbol{\theta})$. The DAEM starts from a vector of random initial parameter values and uses the parameter estimates from the previous step/temperature as the starting values at each subsequent step. Running the DAEM leads to running an EM at each temperature, and thus, the DAEM is slower than a single run of EM. So the DAEM can be thought of as trading time for the procedure's robustness to starting parameter values.

To implement the DAEM in our HMTM problem, notice that $f(\boldsymbol{p},\boldsymbol{S}|\boldsymbol{\theta})$ can be expressed as a product of probabilities and densities raised to the power of indicators of the hidden states, and thus, $f^\gamma(\boldsymbol{p},\boldsymbol{S}|\boldsymbol{\theta})$ has the effect of making all the probabilities and densities raised to the power γ, i.e.,

$$\begin{aligned} f^\gamma(\boldsymbol{p},\boldsymbol{S}|\boldsymbol{\theta}) &= \pi^{\gamma S_1}(1-\pi)^{\gamma(1-S_1)}\prod_{i=2}^{N_T}\omega^{\gamma \mathrm{I}(S_{\rho(i)}=1,S_i=1)}\prod_{i=2}^{N_T}(1-\omega)^{\gamma \mathrm{I}(S_{\rho(i)}=1,S_i=0)} \\ &\quad \prod_{i=1}^{N_T} f_1^{\gamma S_i}(p_i|\alpha,\beta) f_0^{\gamma(1-S_i)}(p_i|\lambda,\alpha_0,\beta_0). \end{aligned}$$

That is, in the E step where we calculate the conditional expectation of S_i and $\mathrm{I}(S_{\rho(i)} = 1, S_i = k)$ under the conditional distribution $f(\boldsymbol{S}|\boldsymbol{p},\boldsymbol{\theta})$, if we use $\pi^\gamma, (1-\pi)^\gamma, \omega^\gamma, (1-\omega)^\gamma$, $f_1^\gamma(p_i|\alpha,\beta)$ and $f_0^\gamma(p_i|\lambda,\alpha_0,\beta_0)$ in the places of $\pi, 1-\pi, \omega, (1-\omega)$, $f_1(p_i|\alpha,\beta)$ and $f_0(p_i|\lambda,\alpha_0,\beta_0)$, we are effectively calculating the expectation under $f_\gamma(\boldsymbol{S}|\boldsymbol{p},\boldsymbol{\theta}^{(t)})$. A similar adaptation of the DAEM has been used in Granat and Donnellan (2002), where the deterministic annealing method was applied to a hidden Markov chain.

2. A SMALL-SCALE SIMULATION STUDY

We reuse the simulation datasets of Liang and Nettleton (2010) to avoid costly computation of permutation-based p-values and MCMC-based posterior probabilities. The simulation datasets of Liang and Nettleton (2010) are based on an old version of the annotation package, i.e., hgu95av2 package version 2.0.1 that contains only 2353 non-empty unique gene sets. In the Section 4 of the main text, we used the current version of the annotation package, i.e., hgu95av2 package version 3.2.3 that includes 8706 gene sets. The change in the number of gene sets reflects the accumulation of more detailed knowledge of gene functions over time. Furthermore, the results here are based on only 20 simulated datasets instead of the 200 datasets per setting in Section 4 of the main text. For simulation details, see Section 4 of Liang and Nettleton (2010).

In this simulation study, the bottom-up procedure was used instead of the global-up procedure. This is because the magnitude of the smallest permutation p-values is limited by the number of possible permutations and the global-up procedure would lead to no rejection in this simulation setting. Other methods compared are the HMM method (Liang and Nettleton 2010), the min-p procedure (Westfall and Young 1993), and the top-down procedure of Meijer and Goeman (2015). The number of rejections and false positives are shown for each method in Table 1. The PDE cutoff for the HMTM and HMM methods is 0.95, while the FWER is controlled at 0.05 level for all other methods.

Table 1: Number of rejections and false positives across 20 simulated datasets for the proposed HMTM method, HMM method, bottom-up procedure, the min-p procedure and top-down procedure. R denotes # of rejections; V denotes # of false positives.

	HMTM		HMM		bottom-up		min-p		top-down	
Dataset	R	V	R	V	R	V	R	V	R	V
1	543	4	495	0	135	0	189	0	93	0
2	509	5	428	1	161	0	195	0	69	0
3	467	5	343	0	180	0	212	0	104	0
4	573	7	436	3	167	0	188	0	72	0
5	516	0	397	0	161	0	166	0	68	0
6	459	18	361	10	148	0	204	0	88	0
7	358	3	340	4	148	0	176	0	86	0
8	358	8	360	9	148	0	159	0	66	0
9	567	25	466	11	182	0	218	0	69	0
10	661	32	585	24	161	0	185	0	50	0
11	318	1	336	2	127	0	130	0	55	0
12	603	54	498	32	182	0	192	0	136	0
13	260	0	260	0	148	0	170	0	64	0
14	338	0	403	0	179	0	200	0	88	0
15	462	26	384	6	182	0	197	0	85	0
16	684	55	562	31	171	0	190	0	180	1
17	381	6	364	6	187	0	207	0	57	0
18	611	30	478	16	133	0	169	0	103	0
19	377	0	274	0	182	0	196	0	92	0
20	395	18	346	3	158	0	191	0	103	0

All three FWER-controlling procedures exhibited excellent performance with regard to type I error control. Only one type I error was made by top-down procedure across all 20 simulated datasets while bottom-up and min-p procedures made no type I error. Among all

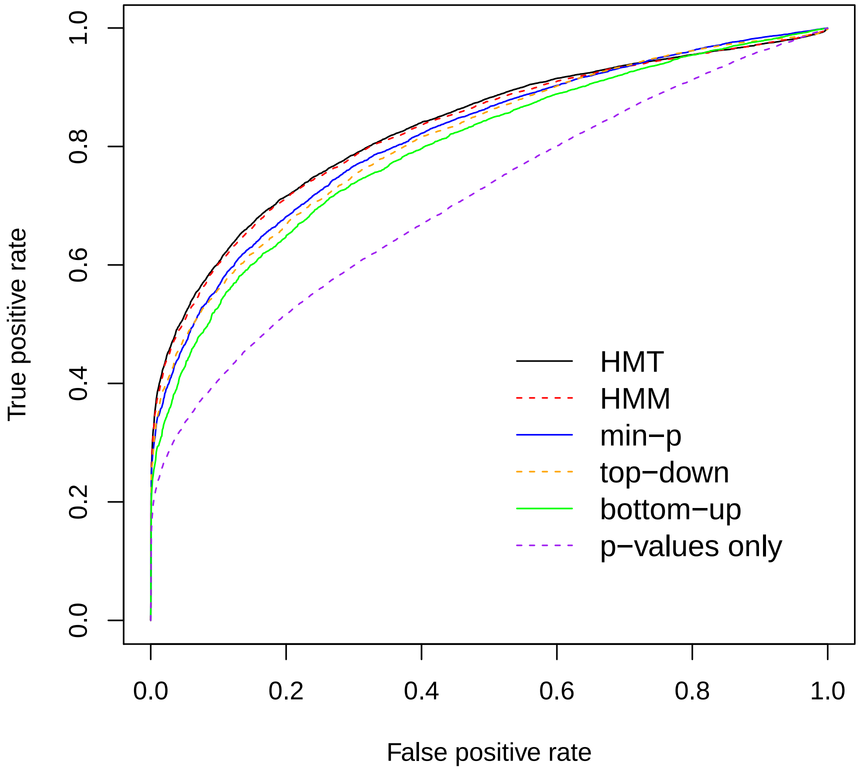

Figure 1: ROC curves.

FWER-controlling procedures, the min-p procedure was the most powerful while the top-down procedure was the least powerful. The HMTM and HMM methods exhibited far more power than either of the FWER-controlling methods, often identifying more than twice as many true positive results at the cost of very few additional false positives.

We also plotted the receiver operating characteristic (ROC) curves in Figure 1 to make a fair comparison among different methods. From Figure 1, the performance of HMTM and HMM methods are the best and are close to each other. The next best are the top-down and min-p procedures, whose performances are similar to each other. The bottom-up procedure is only slightly worse than the top-down and min-p procedures. The p-values only procedure performs the worst because it does not utilize the GO structural information.

REFERENCES

Granat, R. and A. Donnellan (2002). A Hidden Markov Model Based Tool for Geophysical Data Exploration. *Pure and Applied Geophysics 159*(10), 2271–2283.

Liang, K. and D. Nettleton (2010). A hidden markov model approach to testing multiple hypotheses on a tree-transformed gene ontology graph. *Journal of the American Statistical Association 105*(492), 1444–1454.

Meijer, R. J. and J. J. Goeman (2015). A multiple testing method for hypotheses structured in a directed acyclic graph. *Biometrical Journal 57*(1), 123–143.

Ueda, N. and R. Nakano (1998). Deterministic annealing EM algorithm. *Neural Networks 11*(2), 271–282.

Westfall, P. and S. Young (1993). *Resampling-based Multiple Testing: Examples and Methods for p-value Adjustment*. New York, New York: Wiley.
